# Clinical characteristics and prognosis of patients with *Pneumocystis jirovecii* pneumonia without a compromised illness

**DOI:** 10.1371/journal.pone.0246296

**Published:** 2021-02-04

**Authors:** Tae-Ok Kim, Jae-Kyeong Lee, Yong-Soo Kwon, Yu-Il Kim, Sung-Chul Lim, Min-Seok Kim, Bo Gun Kho, Cheol-Kyu Park, In-Jae Oh, Young-Chul Kim, Ha Young Park, Hong-Joon Shin

**Affiliations:** 1 Department of Internal Medicine, Chonnam National University Hospital, Gwangju, Republic of Korea; 2 Chonnam National University Medical School, Gwangju, Republic of Korea; 3 Lung and Esophageal Cancer Clinic, Chonnam National University Hwasun Hospital, Joennam, Republic of Korea; 4 Department of Internal Medicine, Chonnam National University Bitgoeul Hospital, Gwangju, Republic of Korea; Mahidol Oxford Clinical Research Unitl (MORU), THAILAND

## Abstract

**Objective:**

*Pneumocystis jirovecii* pneumonia (PCP) is a fatal respiratory infection, mostly associated with immunocompromised conditions. Several reports have described PCP development in patients who were not immunocompromised, but the clinical course and prognosis of PCP are not well understood. We compared the clinical characteristics and prognoses between patients with and without immunocompromised conditions who developed PCP.

**Methods:**

We retrospectively analyzed patients who had been treated for PCP from three hospitals. We defined immunocompromised (IC) status as following: human immunodeficiency virus (HIV) infection; hematological malignancy; solid organ tumor under chemotherapy; rheumatic disease; medication with immunosuppressive agents. Patients without immunocompromised status were defined as being non-immunocompromised (non-IC).

**Results:**

The IC and non-IC groups comprised 173 and 14 patients. The median ages were 62.0 and 74.0 years in the IC and the non-IC group, respectively. The median interval between admission and anti-PCP treatment was significantly longer for patients in the non-IC group than that for patients in the IC group (7 vs. 2 days). The in-hospital mortality rates were significantly higher for patients in the non-IC group than that for patients in the IC group (71.4% vs. 43.9%; P = 0.047). A longer interval between admission and anti-PCP therapy was associated with increased 90-day mortality rate in patients with PCP (hazard ratio, 1.082; 95% confidence interval, 1.015–1.153; P = 0.016).

**Conclusions:**

Patients with PCP with no predisposing illnesses were older and had higher mortality rates than IC patients with PCP. Delayed anti-PCP treatment was associated with increased 90-day mortality.

## Introduction

*Pneumocystis jirovecii* pneumonia (PCP) is an opportunistic infection caused by *Pneumocystis jirovecii*. Hosts with defective cellular and/or humoral immunity are predisposed to developing PCP. Immunocompromised status such as that caused by human immunodeficiency virus (HIV) infection, hematological malignancies, solid organ tumors under chemotherapy, rheumatic diseases, and medication with immunosuppressive agents is associated with PCP development [1–3]. First-line anti-PCP therapy with trimethoprim/sulfamethoxazole should be initiated as soon as possible after a confirmed diagnosis because PCP is fatal [4,5]. The clinical course is more gradual, and outcomes are better among patients with PCP with HIV infection than in those without HIV [6–11].

Several case reports have described PCP development in patients without immunocompromised illness [12–14]. Although the mechanism of *Pneumocystis* infection in patients without predisposing illness is not clear, poor outcomes are expected if the treatment or diagnosis of PCP are delayed. However, the clinical characteristics and prognosis of PCP in patients without immunocompromised status are not well understood. Therefore, we evaluated the clinical characteristics and prognosis of patients with and without an immunocompromised condition who developed PCP.

## Methods

### Study population and data collection

We retrospectively reviewed the medical charts of patients aged ≥ 18 years who were assessed using *Pneumocystis* polymerase chain reaction (PCR)-based tests between January 2013 and May 2019 at two tertiary referral hospitals and one rheumatic disease-specific hospital. The exclusion criteria were as follows: positive *Pneumocystis* PCR results without anti-PCP treatment; low probability of PCP based on chest computed tomography (CT) images; death or transfer to another hospital within 48 hours of anti-PCP treatment. The following data were analyzed: age; sex; underlying illness; clinical symptoms at the time of presentation; laboratory findings on the day of *Pneumocystis* PCR assay; anti-PCP therapy; interval between admission and receiving anti-PCP treatment; use of mechanical ventilation and high-flow nasal cannula; in-hospital mortality; and 90-day survival while undergoing anti-PCP therapy.

The Institutional Review Board at Chonnam National University Hospital (Gwangju, Republic of Korea) approved the study protocol and permitted the review and publication of our findings, as well as that of information derived from patient records (CNUH-2020-378). Informed consent was waived owing to the retrospective nature of the study, and patient information was rendered innominate before analysis.

### Diagnosis of PCP pneumonia

A diagnosis of PCP was defined as clinical symptoms of fever, dyspnea, or cough, typical radiological PCP findings of bilateral ground glass opacities on chest CT images, positive results in *Pneumocystis* PCR using sputum or bronchoalveolar lavage (BAL) fluid samples, and receiving anti-PCP therapy.

### Immunocompromised status

We defined immunocompromised status as having any of the following: infection with HIV; hematological malignancies; solid organ tumors currently being treated with chemotherapy; rheumatic diseases; and medication with immunosuppressive agents such as glucocorticoids (daily dose equivalent to ≥ 20 mg of prednisolone for ≥ 4 weeks), azathioprine, 6-mercaptopurine, methotrexate, cyclosporine, or tacrolimus, and biologics such as infliximab, adalimumab, golimumab, or vedolizumab). Patients with at least one of the criteria described above were referred to as being immunocompromised (IC) and were sub-grouped based on whether or not they were also infected with HIV. Patients who were negative for the above criteria were defined as being non-immunocompromised (non-IC).

### Statistical analysis

All data are reported as medians with interquartile ranges (IQR) or numbers (%). Continuous variables were analyzed using Mann‒Whitney U-tests for two groups, or Kruskal‒Wallis tests for three groups. Categorical variables were analyzed using Chi-square or Fisher exact tests. Comparisons among three groups were corrected *post hoc* using Bonferroni correction. Factors associated with in-hospital mortality were selected by univariate analysis with logistic regression. Subsequent multivariate logistic regression analysis included variables with P < 0.1 in univariate analysis using a backward method. Ninety-day survival was estimated by Kaplan‒Meier analyses. Factors associated with 90-day mortality were identified using Cox-regression analysis that included variables with P < 0.1 in univariate analysis in the backward method. All data were statistically analyzed using SPSS version 22.0 (IBM Corp., Armonk, NY, USA), and values with P < 0.05 were considered statistically significant.

## Results

**Fig 1:**
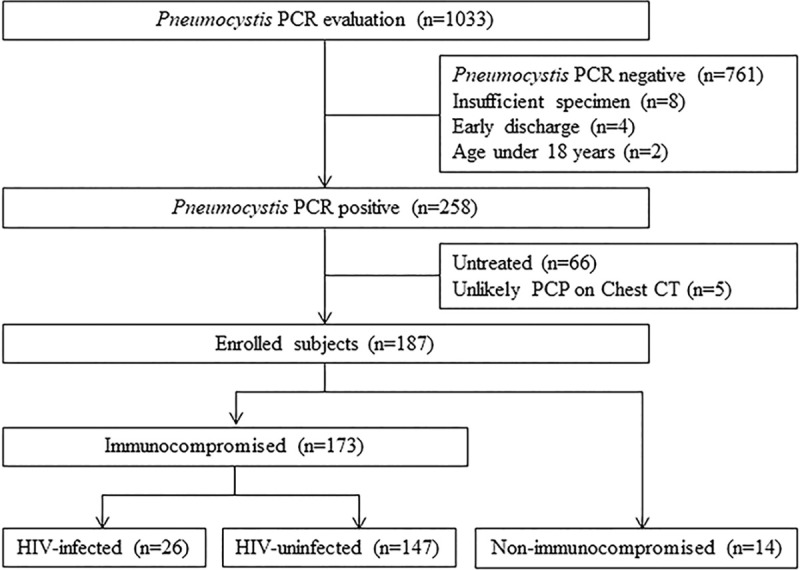
Study flowchart. CT, computed tomography; HIV, human immunodeficiency virus; PCP, *Pneumocystis jirovecii* pneumonia; PCR, polymerase chain reaction.

Among the 1,033 patients who underwent *Pneumocystis* PCR tests during the study period, 258 were positive (Fig 1). Of these 258 patients, 66 did not receive anti-PCP therapy. Five patients did not have typical chest CT findings associated with PCP infection. Therefore, a total of 187 patients who received anti-PCP treatment were enrolled in this study. Of these, 173 patients were in the IC group and 14 were in the non-IC group. In the IC group, 26 patients were HIV-infected and 147 were HIV-uninfected.

### Patient characteristics

Table 1 summarizes patient baseline demographics and clinical characteristics. The respective median ages were 44.5, 64.0, and 74.0 years in the HIV-infected, HIV-uninfected IC, and non-IC groups. All HIV-infected patients were male; however, men represented 67.3% of the HIV-uninfected IC group and 42.9% of non-IC group. Hematological malignancies were the most prevalent in the HIV-uninfected IC group, followed by use of medication with immunosuppressive agents. Ischemic heart disease was significantly more prevalent in the non-IC group, than that in the other two groups. The HIV-infected group had the lowest alveolar-arterial oxygen pressure difference (AaDO_2_) levels and highest PaO_2_/FiO_2_ (P/F) ratio among all groups. However, the AaDO_2_ and P/F ratios did not significantly differ between HIV-uninfected IC group and the non-IC group. Almost all patients received initial anti-PCP treatment with trimethoprim/sulfamethoxazole. The intervals between admission and anti-PCP treatment differed among the three groups (medians 1 vs. 3 vs. 7 days, respectively). *Post hoc* analysis showed that the interval between admission and receiving anti-PCP treatment was significantly longer for the non-IC group than that for the HIV-infected group. Adjuvant glucocorticoid therapy was administered to significantly fewer patients in the HIV-infected group than that in the other two groups. The in-hospital mortality rate was the highest in the non-IC group. *Post hoc* analysis showed that in-hospital mortality rates significantly differed between the HIV-infected and the non-IC group.

**Table 1:** Comparison of clinical characteristics between the three groups.

Variables	HIV-infected (n = 26)	HIV-uninfected IC (n = 147)	Non-IC (n = 14)	P
Median age, y (range)	44.5 (37.5–54.2)	64.0 (57.0–72.0)	74.0 (71.2–80.2)	<0.000
Male	26 (100%)	99 (67.3%)	6 (42.9%)	<0.000
Hematological malignancy	―	80 (54.4%)	―	
Solid organ tumor	―	21 (14.3%)	―	
Rheumatic disease	―	21 (14.3%)	―	
Medication with immunosuppressive agents	―	25 (17.0%)	―	
Hypertension	2 (7.7%)	56 (38.1%)	4 (28.6%)	0.009
Diabetes	1 (3.8%)	37 (25.2%)	2 (14.3%)	0.040
Chronic obstructive lung disease	1 (3.8%)	6 (4.1%)	1 (7.1%)	0.858
Interstitial lung disease	1 (3.8%)	9 (6.1%)	3 (21.4%)	0.079
Ischemic heart disease	1 (3.8%)	10 (6.8%)	7 (50.0%)	<0.000
Chronic kidney disease	0 (0%)	16 (10.9%)	3 (21.4%)	0.083
Chronic liver disease	1 (3.8%)	13 (8.8%)	1 (7.1%)	0.683
Cough	14 (53.8%)	59 (40.1%)	10 (71.4%)	0.046
Fever	9 (34.6%)	68 (46.3%)	6 (42.9%)	0.541
Dyspnea	16 (61.5%)	80 (54.4%)	12 (85.7%)	0.070
BAL fluid	11 (42.3%)	14 (9.5%)	3 (21.4%)	<0.000
WBC (*10^3^/μL)	6.7 (3.1–8.3)	7.7 (2.9–11.9)	12.0 (9.4–14.6)	0.024
CRP (mg/dL)	3.0 (0.8–3.0)	12.1 (8.3–19.3)	11.6 (4.2–18.4)	<0.000
LDH (U/L)	778 (640–1036)	809 (541–1194)	1078 (849–1574)	0.134
AaDO_2_ (mmHg)	44 (20–119)	393 (109–464)	474 (75–488)	<0.000
P/F ratio (mmHg)	147 (86–159)	46 (36–104)	45 (38–94)	<0.000
Initial treatment				0.760
Trimethoprim/sulfamethoxazole	26 (100%)	145 (98.6%)	14 (100%)	
Primaquine + clindamycin	0	2 (1.4%)	0	
Interval between admission and anti-PCP treatment, days	1.0 (0–4.2)	3.0 (0–7.0)	7.0 (2.0–10.0)	0.014
Glucocorticoids	13 (50.0%)	125 (85.0%)	12 (85.7%)	<0.000
Antibiotics	0.5 (0–2.0)	2.0 (1.0–2.0)	2.0 (2.0–2.0)	<0.000
Mechanical ventilator support	5 (19.2%)	57 (38.8%)	9 (64.3%)	0.018
High-flow nasal cannula	4 (15.4%)	35 (24.0%)	5 (35.7%)	0.346
In-hospital mortality	4 (15.4%)	72 (49.0%)	10 (71.4%)	0.001

Data are presented as median (interquartile range) or number (%). AaDO_2_, alveolar-arterial oxygen pressure difference; BAL, bronchoalveolar lavage; CRP, C-reactive protein; HIV, human immunodeficiency virus; IC, immunocompromised; LDH, lactate dehydrogenase; PCP, *Pneumocystis jirovecii* pneumonia; P/F, PaO_2_/FiO_2_; WBC, white blood cells.

### Prognostic factors associated with in-hospital mortality among patients with PCP

Univariate logistic regression analysis revealed that advanced age, HIV infection, interstitial lung disease, dyspnea, higher AaDO_2_, and lower P/F ratios were significantly associated with in-hospital mortality among patients with PCP (Table 2). Multivariate analysis revealed advanced age, higher AaDO_2,_ and dyspnea to be associated with increased in-hospital mortality rates among patients with PCP.

**Table 2:** Prognostic factors associated with in-hospital mortality.

Univariate analysis			
Variables	OR	95% CI	P
Age	1.043	1.019–1.068	<0.000
Male	0.717	0.383–1.343	0.299
IC group with HIV	0.175	0.058–0.531	0.002
IC group without HIV	1.783	0.863–3.684	0.118
Non-IC group	3.191	0.963–10.570	0.058
Cardiovascular disease	1.195	0.452–3.159	0.720
Interstitial lung disease	0.138	0.030–0.640	0.011
Dyspnea	2.803	1.526–5.148	0.001
C-reactive protein	1.001	0.990–1.011	0.870
AaDO_2_	1.003	1.001–1.005	0.007
P/F ratio	0.988	0.980–0.997	0.006
Lactate dehydrogenase	1.000	1.000–1.000	0.831
Interval between admission and anti-PCP treatment	1.044	0.978–1.116	0.195
**Multivariate analysis**			
Variables	OR	95% CI	P
Age	1.060	1.024–1.104	0.001
AaDO2	1.002	1.000–0.005	0.016
Dyspnea	3.625	1.052–12.489	0.041

AaDO_2_, alveolar-arterial oxygen pressure difference; CI, confidence interval; HIV, human immunodeficiency virus; IC, immunocompromised; OR, odds ratio; PCP, *Pneumocystis jirovecii* pneumonia; P/F, PaO_2_/FiO_2_.

### Prognostic factors associated with 90-day survival among patients with PCP

**Fig 2:**
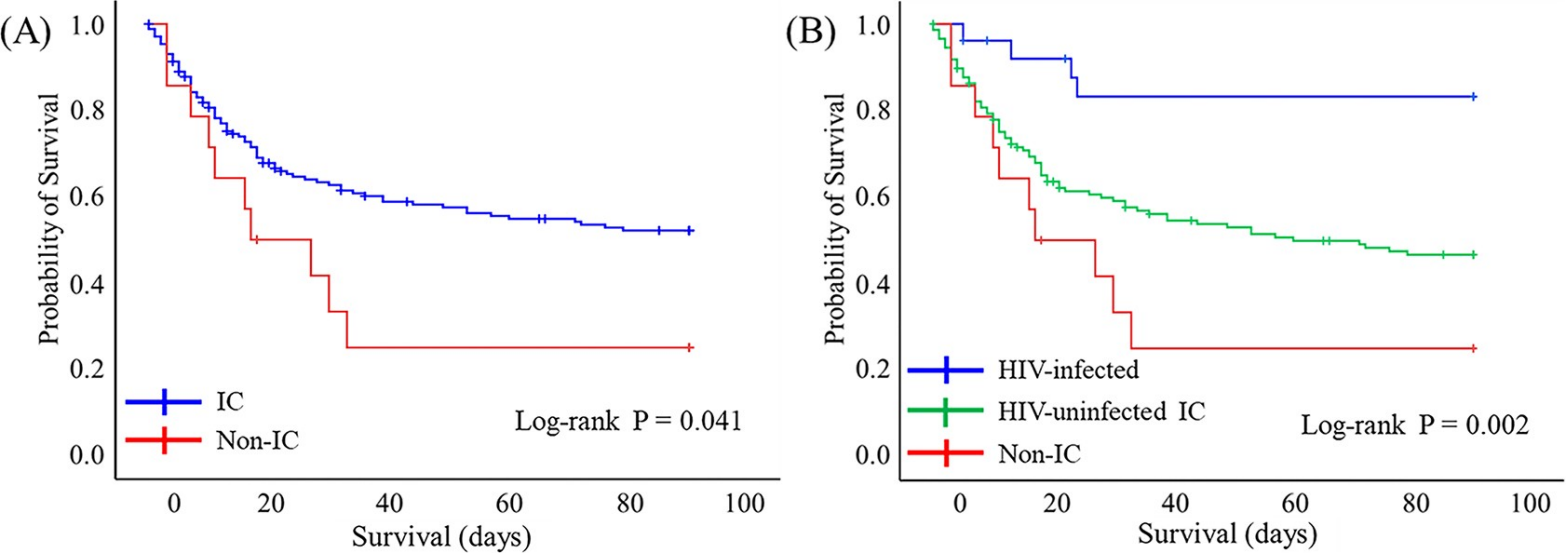
Kaplan‒Meier curves show 90-day survival after initiating the anti-PCP treatment. (A) IC and non-IC groups, (B) IC groups with and without HIV, and non-IC. HIV, human immunodeficiency virus; IC, immunocompromised; PCP, *Pneumocystis jirovecii* pneumonia.

Fig 2A shows that 90-day survival after initiating the anti-PCP therapy was significantly worse in the non-IC group than that in the IC group (P = 0.041, log-rank test). HIV-infected group had the best 90-day survival rate whereas the non-IC group and the HIV-uninfected IC group had the lowest and intermediate 90-day survival rates, respectively (P = 0.002, log-rank test; Fig 2B). Cox-regression analysis using the non-IC group as a reference revealed lower 90-day mortality rates in the HIV-infected group than that in the non-IC group (hazard ratio [HR], 0.143; 95% confidence interval [CI], 0.045–0.456; P = 0.001]. However, 90-day mortality rates did not significantly differ between the HIV-uninfected IC group and the non-IC group (HR, 0.591; 95% CI, 0.305–1.146; P = 0.119).

Univariate analysis revealed the 90-day mortality to be associated with advanced age, HIV infection, interstitial lung disease, dyspnea, a higher AaDO_2_, a lower P/F ratio, and a longer interval between admission and receiving anti-PCP treatment among patients with PCP (Table 3). Multivariate analysis revealed the 90-day mortality rates to be associated with advanced age, interstitial lung disease, a lower P/F ratio, and longer interval between admission and anti-PCP treatment among patients with PCP.

**Table 3:** Prognostic factors associated with 90-day mortality after initiating anti-PCP treatment.

**Univariate analysis**			
Variables	HR	95% CI	P
Age	1.305	1.018–1.052	<0.000
Male	0.805	0.518–1.252	0.805
HIV-infected group	0.230	0.084–0.627	0.004
HIV-uninfected IC group	1.604	0.906–2.841	0.105
Non-IC group	1.957	1.011–3.789	0.046
Cardiovascular disease	1.163	0.584–2.318	0.667
Interstitial lung disease	3.195	1.682–6.069	<0.000
Dyspnea	2.301	1.458–3.631	<0.000
C-reactive protein	1.001	0.995–1.006	0.861
AaDO_2_	1.002	1.000–1.003	0.010
P/F ratio	0.993	0.987–0.998	0.014
Lactate dehydrogenase	1.000	1.000–1.000	0.834
Interval between admission and anti-PCP treatment	1.049	1.002–1.098	0.039
**Multivariate analysis**			
Variables	HR	95% CI	P
Age	1.060	1.009–1.061	0.008
Interstitial lung disease	2.487	1.183–5.228	0.016
P/F ratio	0.992	0.982–0.999	0.036
Duration between admission and anti-PCP treatment, days	1.082	1.015–1.153	0.016

AaDO_2_, alveolar-arterial oxygen pressure difference; CI, confidence interval; HIV, human immunodeficiency virus; HR, hazard ratio; IC, immunocompromised; PCP, *Pneumocystis jirovecii* pneumonia; P/F, PaO_2_/FiO_2_.

### Subgroup analysis of the non-IC group

Baseline characteristics did not significantly differ between survivors (n = 4) and non-survivors (n = 10) in the non-IC group (Table 4). The median interval between admission and anti-PCP treatment was longer for those who died than for those who survived (9 vs. 6 days, respectively), but the difference was not significant. Univariate logistic regression analysis did not identify the risk factors associated with in-hospital mortality in the non-IC group (Table 5).

**Table 4:** Comparison of the clinical characteristics between survivors and non-survivors in the non-immunocompromised group.

Variables	Survivors (n = 4)	Non-survivors (n = 10)	P
Mean age y (range)	76.0 (37.5–80.7)	74.0 (71.2–79.7)	0.887
Male	3 (75.0%)	3 (30.0%)	0.245
Hypertension	1 (25.0%)	3 (30.0%)	1.000
Diabetes	0 (0%)	2 (20.0%)	1.000
COPD	0 (0%)	1 (10.0%)	1.000
Cardiovascular disease	3 (75.0%)	4 (40.0%)	0.559
Interstitial lung disease	0 (0%)	3 (30.0%)	0.505
Chronic kidney disease	2 (50.0%)	1 (10.0%)	0.176
Chronic liver disease	0 (0%)	1 (10.0%)	1.000
Cough	2 (50%)	8 (80%)	0.520
Sputum	1 (25%)	5 (50%)	0.580
Fever	2 (50%)	4 (40%)	1.000
Dyspnea	2 (50%)	10 (100%)	1.000
BAL sample	3 (75.0%)	2 (20.0%)	1.000
WBC (*10^3^/μL)	12.0 (11.2–13.8)	11.3 (7.1–15.8)	0.777
CRP (mg/dL)	9.0 (4.8–13.5)	14.2 (3.7–20.8)	0.671
LDH (U/L)	908 (498–1332)	1293 (870–1708)	0.157
Duration between admission and anti-PCP treatment, days	6.0 (2.0–9.2)	9.0 (2.0–10.0)	0.825
Glucocorticoids	3 (75.0%)	9 (90.0%)	0.505
Mechanical ventilator support	2 (50.0%)	7 (70%)	0.580
HFNC support	2 (50.0%)	3 (30%)	0.580

Data are presented as median (interquartile range) or number (%). BAL, bronchoalveolar lavage; COPD, chronic obstructive pulmonary disease; CRP, C-reactive protein; HFNC, high-flow nasal cannula; HIV, human immunodeficiency virus; IC, immunocompromised; LDH, lactate dehydrogenase; PCP, *Pneumocystis jirovecii* pneumonia; WBC, white blood cells.

**Table 5:** Prognostic factors associated with in-hospital death in the non-immunocompromised group.

Variables	OR	95% CI	P
Age	1.047	0.958–1.144	0.314
Male	0.143	0.010–1.995	0.148
Cardiovascular disease	0.222	0.017–2.970	0.256
White blood cell	1.000	1.000–1.000	0.640
C-reactive protein	1.070	0.911–1.258	0.408
Lactate dehydrogenase	1.002	0.999–1.005	0.201
AaDO_2_	1.000	0.994–1.007	0.892
P/F ratio	1.008	0.971–1.047	0.677
Duration between admission and anti-PCP treatment	1.044	0.978–1.116	0.195

AaDO_2_, alveolar-arterial oxygen pressure difference; CI, confidence interval; OR, odds ratio; PCP, *Pneumocystis jirovecii* pneumonia; P/F, PaO_2_/FiO_2_.

## Discussion

To the best of our knowledge, this study is the first to describe the clinical characteristics and prognosis of patients with PCP who are not immunocompromised by the occurrence of another illness. Patients with PCP without a predisposing illness were old in age and had delayed anti-PCP treatment. These patients also required mechanical ventilation more frequently, and had higher in-hospital and 90-day mortality rates than immunocompromised patients. Delayed anti-PCP treatment in patients with PCP was associated with increased 90-day mortality rates.

The gold standard for diagnosing PCP in immunocompromised patients is the detection of *Pneumocystis* in the BAL fluid or induced sputum samples using microscopy [15]. However, this method has low sensitivity and negative predictive value [15]. Recently, the *Pneumocystis* PCR assay using BAL fluid or induced sputum has been widely used [15]. Although this method cannot distinguish true infection from false positive results or colonization, it has high sensitivity and negative predictive value [16]. We assayed BAL fluid (n = 3), tracheal aspirate (n = 6), and expectorated sputum (n = 5) samples from the non-IC group using *Pneumocystis* PCR. BAL fluid has been identified as the optimal sample for *Pneumocystis* PCR assay; however, tracheal aspirates and expectorated sputum can also be assayed in life-threatening situations [17]. Quantitative PCR assays have been combined with immunofluorescence-based tests, and serum β-D-glucan assays to distinguish PCP from colonization [18,19]. However, the cutoff values for true infection in these combined tests have not been conclusively identified. Pneumocystis colonization could not be ruled completely out in the present study; the Pneumocystis-positive status determined by PCR along with respiratory symptoms, such as fever, dyspnea, and cough, and bilateral ground glass opacities in chest CT provided sufficient evidence for Pneumocystis infection. PCP is a major opportunistic infection in immunocompromised patients infected with HIV. However, the incidence of PCP has increased in patients who are not immunocompromised by HIV [20]. Predisposing factors for the development of PCP in patients without HIV include glucocorticoid medication, hematological malignancies, and use of various immunosuppressive agents [1–3]. However, several case reports have described patients who developed PCP in the absence of immunocompromised illness [12–14,21]. Jacobs *et al*. described five elderly PCP patients without predisposing illnesses [22]. Cano *et al*. also described five young patients aged 30–55 years who developed PCP without being immunocompromised by the presence of another illness [23]. The present study found no evidence of HIV infection in the non-IC group or of disease that compromised the immune system at the time of initial evaluation.

The clinical characteristics differed among the non-IC group and the IC groups with and without HIV. The IC group with HIV had the youngest patients, whereas the non-IC group was represented by the oldest patients. A previous study found that patients with PCP and without HIV were older than those with PCP together with HIV [8,24,25]. The present finding that the non-IC group contained patients who were older than those in the IC group without HIV indicated that PCP can occur without predisposing illness in the elderly. Similar to the present findings, the five patients with PCP reported by Jacobs *et al*. were aged between 66 and 78 years and exhibited no predisposing factors [22]. However, Cano *et al*. described 5 patients aged between 30 and 55 years with PCP who also had no predisposing illness for PCP [23]. Further studies are needed to determine whether advanced age is a risk factor for development of PCP in individuals without predisposing illnesses. Among the non-IC group in the present study, 50% of the patients had ischemic heart disease. Two (40%) of the five patients with PCP described by Jacobs *et al*., had congestive heart failure [22]. The clinical course was far worse among the non-IC group than that in the IC group with HIV. The AaDO_2_ value was higher, the P/F ratio was lower and the frequency of mechanical ventilator support was higher in the non-IC group than that in the IC group with HIV. The clinical course did not significantly differ between the non-IC group and the IC group without HIV.

In-hospital and 90-day mortality rates were the lowest in the HIV-infected group in the present study. Previous studies have found that survival was better for PCP patients with HIV than that for patients with PCP without HIV [8,24–26]. Several factors can explain the better prognosis of the HIV-infected patients with PCP than that of HIV-uninfected patients with PCP [8,24–27]. Patients with PCP who had HIV as a comorbidity were younger [8,24,25]. Patients with PCP with HIV required mechanical ventilation for respiratory failure less frequently than that patients with PCP who without HIV infection as a comorbidity [8]. Neutrophil counts were higher in the BAL fluid from patients with PCP without HIV than those from PCP patients with HIV [24]. Therefore, lung injury was considered more severe in patients with PCP without HIV [24]. In addition, adjuvant glucocorticoids can reduce mortality among PCP patients with HIV, whereas the effect has not been proven in patients with PCP without HIV [28–30]. As with previous findings, the present study found worse 90-day mortality rates in the HIV-uninfected IC group than those in HIV-infected group (HR, 4.145; 95% CI, 1.515–11.343; P = 0.001). The present study also found worse 90-day mortality rates for the non-IC group than that for the HIV-infected group (HR, 7.017; 95% CI, 2.194–22.440; P = 0.001). Advanced age was associated with high mortality rates among patients with PCP in this study. The non-IC group contained the oldest patients among the three groups, indicating that advanced age is closely associated with the high mortality rate in this group. A previous study found the mortality rate to be 60% among older patients with PCP and no predisposing illness [22]. Whereas, all young patients without predisposing factors who had PCP survived [23]. The in-hospital mortality rate was higher among patients in the non-IC group than that in patients in the HIV-uninfected IC group (71.4% vs. 49.0%.), but the difference was not significant as per *post hoc* analysis. Further multicenter-prospective studies are needed.

Longer interval between admission and receiving anti-PCP treatment was associated with higher 90-day mortality rates. A previous study has shown that patients with PCP but not HIV receive anti-PCP treatment later than those with PCP together with HIV [31,32]. Furthermore, delayed anti-PCP treatment is a risk factor for mortality [32]. The present study found that the non-IC group received the anti-PCP treatment at hospitals at a median of 6 days later than the IC group with HIV. The interval between admission and anti-PCP treatment was 3 days longer among patients in the non-IC group who died than in those who survived, but the difference was not significant. Therefore, if clinical symptoms and imaging findings indicate a reasonable probability of PCP, then a diagnostic evaluation and early treatment are necessary, regardless of the immunocompromised status.

The present study has several limitations. First, the nature of this study is retrospective design. Second, the number of patients enrolled in this study was small, despite data were collected for 6 years from three hospitals. Because PCP is rare disease, further multicenter-prospective studies are needed. Third, the possibility of *Pneumocystis* colonization could not be completely excluded. Tests such as quantitative PCR, immunofluorescence assays, or serum β-D-glucan measurements might help distinguish true *Pneumocystis* infection from colonization, but such tests were not available at any of the three hospitals [18,19,33]. Fourth, we did not measure immunoglobulin classes and T-cell subpopulations in all patients without IC. Therefore, the possibility of hidden immunodeficiency conditions could not be completely excluded.

## Conclusion

Patients with PCP who had no predisposing illness were older and had higher mortality rates than patients with PCP who were immunocompromised. Delayed anti-PCP treatment was associated with increased 90-day mortality rates. Therefore, if clinical symptoms and imaging findings point to a reasonable likelihood of PCP, then diagnostic evaluations and early treatment for PCP should be undertaken as soon as possible, regardless of the immunocompromised status.

## Supporting information

S1 Dataset (XLSX)
